# Multiple autophosphorylations significantly enhance the endoribonuclease activity of human inositol requiring enzyme 1α

**DOI:** 10.1186/1471-2091-15-3

**Published:** 2014-02-13

**Authors:** Daniel Itzhak, Michael Bright, Peter McAndrew, Amin Mirza, Yvette Newbatt, Jade Strover, Marcella Widya, Andrew Thompson, Gareth Morgan, Ian Collins, Faith Davies

**Affiliations:** 1 From the Division of Cancer Therapeutics, Institute of Cancer Research, Sutton, Surrey SM2 5NG, UK; 2 Division of Molecular Pathology, Institute of Cancer Research, Sutton, Surrey SM2 5NG, UK; 3 Proteomics Core Facility, Institute of Cancer Research, London SW3 6JB, UK

**Keywords:** Endoplasmic reticulum stress, Enzyme mechanisms, ER stress, Mass spectrometry (MS), Multiple myeloma, Ribonuclease, Unfolded protein response, IRE1, Autophosphorylation

## Abstract

**Background:**

Endoplasmic reticulum stress, caused by the presence of misfolded proteins, activates the stress sensor inositol-requiring enzyme 1α (IRE1α). The resulting increase in IRE1α RNase activity causes sequence-specific cleavage of X-box binding protein 1 (XBP1) mRNA, resulting in upregulation of the unfolded protein response and cellular adaptation to stress. The precise mechanism of human IRE1α activation is currently unclear. The role of IRE1α kinase activity is disputed, as results from the generation of various kinase-inactivating mutations in either yeast or human cells are discordant. Kinase activity can also be made redundant by small molecules which bind the ATP binding site. We set out to uncover a role for IRE1α kinase activity using wild-type cytosolic protein constructs.

**Results:**

We show that concentration-dependent oligomerisation is sufficient to cause IRE1α cytosolic domain RNase activity in vitro. We demonstrate a role for the kinase activity by showing that autophosphorylation enhances RNase activity. Inclusion of the IRE1α linker domain in protein constructs allows hyperphosphorylation and further enhancement of RNase activity, highlighting the importance of kinase activity. We show that IRE1α phosphorylation status correlates with an increased propensity to form oligomeric complexes and that forced dimerisation causes great enhancement in RNase activity. In addition we demonstrate that even when IRE1α is forced to dimerise, by a GST-tag, phospho-enhancement of activity is still observed.

**Conclusions:**

Taken together these experiments support the hypothesis that phosphorylation is important in modulating IRE1α RNase activity which is achieved by increasing the propensity of IRE1α to dimerise. This work supports the development of IRE1α kinase inhibitors for use in the treatment of secretory cancers.

## Background

Inositol-requiring enzyme 1α (IRE1α) is an endoplasmic reticulum (ER) stress sensor activated by the accumulation of unfolded proteins. IRE1α activation results in the production of XBP1s, a transcription factor, leading to increased expression of genes involved in membrane synthesis, protein folding and protein degradation [1-3], termed the unfolded protein response (UPR) [4]. This response enables cells to adapt to ER stress caused for example by an increased protein load [5]. The UPR has recently been shown to play an important role in cancer biology, particularly in tumours with a secretory cell origin [6,7]. An example of this is multiple myeloma, a malignancy of plasma cells, which produce large quantities of an immunoglobulin or paraprotein. These cells are addicted to the UPR to manage the high protein production which would otherwise be toxic. Thus, IRE1α activity and XBP1s production are thought to be critical to the development and maintenance of the myeloma clone [3,8,9] and have therefore been proposed as possible therapeutic targets [10].

IRE1α consists of a lumenal stress-sensing domain, transmembrane helix, cytosolic linker domain followed by kinase and RNase domains [11]. Accumulation of unfolded proteins in the ER lumen leads to release of binding protein (BiP) from the IRE1α lumenal domain allowing dimerisation [12]. In yeast, direct binding of unfolded protein to Ire1 is additionally required for oligomerisation [13] though this is not thought to occur with human IRE1α [14]. The resulting oligomerisation enables trans-autophosphorylation of the Ire1/IRE1α cytosolic domain which activates the RNase [12,15], whose active site is generated by dimerisation [16].

A number of pieces of data support a model where oligomerisation is essential and kinase activity is dispensable for the RNase activity. The requirement for autophosphorylation in yeast and human IRE1α can be made redundant by small molecules which bind the kinase ATP site in lumenal domain deleted Ire1 or kinase mutated Ire1 [17-19]. *In vitro* studies have also demonstrated that inclusion of the Ire1 linker domain permits the formation of higher-order oligomeric structures and increased activity, even when the kinase is mutated [18]. Although the validity of this model is debated as kinase inactivating mutations may or may not lack activity [20,21]. Moreover, the linker domain, whose presence is required for oligomerisation, is not conserved between yeast and human, yet human IRE1α is also thought to form higher-order oligomers [22].

In this work we sought to address the role of the kinase and the linker domain in human IRE1α. Experiments expressing IRE1α in cells are fraught with difficulty due to spontaneous activation when overexpressed [23]. We therefore chose not to use further kinase mutants to study the role of the human kinase, instead, our approach involved using purified IRE1α cytosolic domain with phosphatase incubation or ATP incubation to simulate the dephosphorylated and autophosphorylated states respectively. We show that dimerisation/oligomerisation of IRE1α is sufficient for RNase activity but that phosphorylation of the IRE1α cytosolic domain enables RNase activity at lower concentrations. We also show that inclusion of the linker domain enables hyperphosphorylation of human IRE1α which further reduces the concentration at which the RNase is active.

## Results and discussion

### Concentration dependent oligomerisation of human IRE1α activates RNase activity in vitro

Previously, partially dephosphorylated or kinase dead yeast Ire1 has been shown to have an activated RNase following oligomerisation *in vitro*[18]. Human phosphorylated IRE1α has also been shown to have an activated RNase following oligomerisation at high concentrations *in vitro* and *in vivo*[19,22]. We sought to determine if fully dephosphorylated human IRE1α could become RNase active following oligomerisation *in vitro*.

**Figure 1:**
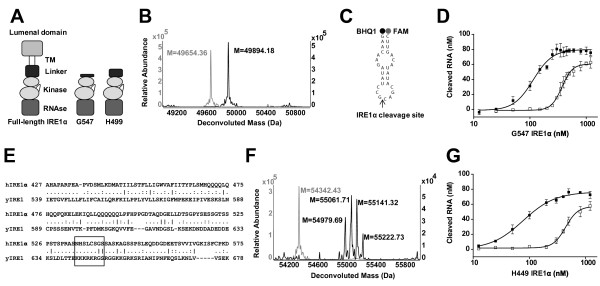
**Phosphorylation enhances activity of IRE1α *in vitro*. (A)** Schematic of the truncated G547 and H499 IRE1α construct compared to the full-length protein. **(B)** Deconvoluted mass spectra of lambda phosphatase-treated G547 IRE1α produced in insect cells (grey) and after incubation with Mg/ATP *in vitro* (black) show the addition of 3 phosphates due to autophosphorylation. **(C)** Schematic of the *in silico* designed stembulge RNA containing the XBP-1 splice site labelled 5’ with fluorescein (FAM) and 3’ with Black-Hole Quencher 1 (BHQ1) whose fluorescence quenching is alleviated upon cleavage. **(D)** 90 nM RNA in **C** was incubated with increasing concentrations of dephosphorylated IRE1α (open squares, EC_50_ = 369 nM ) or phosphorylated IRE1α (filled squares, EC_50_ = 114 nM) for 30 minutes at 30˚C. Error bars S.E.M of 3 independent experiments. **(E)** Linker regions of human and yeast IRE1. The linker domain is defined by the first residue after the transmembrane domain and the last residue before the kinase domain (human P465-S570, yeast Q556-L673). Human IRE1α linker domain is more Ser/Thr-rich 26/106aa (24.5%) than yeast Ire1 16/118aa (13.6%) linker domain. The lysine-rich region of the yeast linker domain is boxed. Full-length human IRE1α and yeast IRE1 sequences were aligned using EMBOSS stretcher [http://www.ebi.ac.uk/Tools/psa/emboss_stretcher/]. **(F)** Deconvoluted mass spectra of lambda phosphatase-treated H499 IRE1α produced in insect cells (grey) and after incubation with Mg/ATP *in vitro* (black) show the addition of multiple phosphates (8–11) due to autophosphorylation. **(G)** As in **D**, dephosphorylated H499 IRE1α (open squares, EC_50_ = 440 nM), autophosphorylated H499 IRE1α (filled squares, EC_50_ = 77 nM).

An IRE1α kinase and RNase domain construct encompassing residues G547-L977, designated G547 IRE1α, which retains kinase autophosphorylation activity [24] was produced in insect cells (Figure 1A) and dephosphorylated by treatment with λ-phosphatase. Dephosphorylation was confirmed by western blot analysis using an antibody directed at phospho-serine 724 [24,25] (Additional file 1: Figure S1), and by mass spectrometry of intact protein (Figure 1B).

To test the RNase activity of fully dephosphorylated IRE1α, an *in vitro* FRET-derepression assay was developed [10] where cleavage of an internally quenched XBP1 splice site mimic leads to increased fluorescence (Figure 1C). Increasing concentrations of dephosphorylated G547 IRE1α were incubated with the XBP1 splice site mimic and fluorescence was measured. Below 150 nM no cleavage was detected but increasing G547 IRE1α concentrations resulted in significant cleavage with an EC_50_ of 369 nM and maximal activity observed at 800 nM (Figure 1D). These results demonstrate, for the first time, that oligomerisation can drive RNase activity of human IRE1α cytosolic domain in the absence of phosphorylation.

### Autophosphorylation enhances RNase activity

In order to determine the role of phosphorylation in regulating RNase activity dephosphorylated G547 IRE1α was incubated with Mg/ATP to induce autophosphorylation. Phosphorylation was confirmed by western blotting (Additional file 1: Figure S1) and mass spectrometry (Figure 1B) which showed a tri-phosphorylated protein as the major species. Autophosphorylated G547 IRE1α was then tested in the FRET-derepression assay at a range of concentrations (Figure 1D). RNase activity was observed at concentrations as low as 50 nM, with maximal activity at 350 nM and an EC_50_ of 114 nM. Thus the autophosphorylated form of G547 IRE1α required substantially lower protein concentration than the dephosphorylated form to activate the RNase by oligomerisation indicating that autophosphorylation enhances RNase activity. Indeed, RNase activity is enhanced to the extent that there is a significant concentration window where the phosphorylated form has activity whilst the dephosphorylated form remains inactive.

### Extension of the linker domain further enhances phosphorylation-dependent RNase activity in vitro

The lysine rich region of the linker domain, critical for enhancement of yeast Ire1 endonuclease activity [18], is not conserved in human IRE1α (Figure 1E). To investigate the functional role of the linker region in human IRE1α, a construct containing an additional 48 amino acids of the linker domain (H499-L977, designated H499 IRE1α) was produced in insect cells. Both dephosphorylated and autophosphorylated proteins were produced to assess their activity. The mass spectrum of λ-phosphatase treated H499 IRE1α confirmed the absence of phosphate groups, while incubation with Mg/ATP resulted in the appearance of multiple polyphosphorylated forms (8 to 11 phosphorylations) (Figure 1F, (Additional file 1: Figure S1). The presence of the extra phosphorylations compared to G547 IRE1α is consistent with the serine/threonine-rich sequence of the linker domain (Figure 1E).

Autophosphorylated H499 IRE1α had enhanced RNase activity compared to autophosphorylated G547 IRE1α with activity observed at concentrations as low as 25 nM, maximal activity at 350 nM and an EC_50_ of 77 nM (Figure 1D and G). The dephosphorylated H499 IRE1α construct had no activity below concentrations of 200 nM, maximal activity at 800 nM with an EC_50_ of 440 nM (Figure 1G). This data demonstrates that extension of the linker enables activation of phosphorylated IRE1α at lower concentrations. Interestingly, despite a lack of conservation between human and yeast linker domains, both play a role in enhancing RNase activity of Ire1/IRE1α suggesting that the linker domain is important in regulated RNase activity [18].

### GST-mediated dimerisation of IRE1α enhances RNase activity in vitro

**Figure 2:**
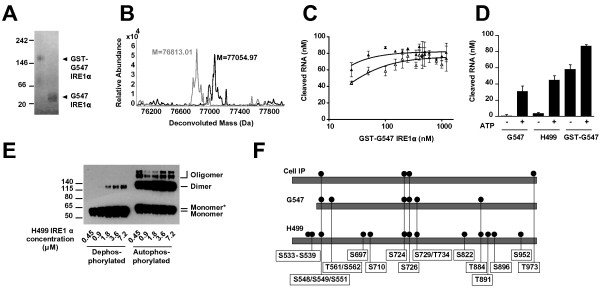
**Dimerisation of human IRE1α enhances ribonuclease activity. (A)** Native polyacrylamide gel showing that GST-G547 IRE1α (77 kDa) migrates as dimers whereas G547 IRE1α (49.6 kDa) migrates as monomers. **(B)** Intact protein mass spectra of lambda phosphatase treated GST-G547 IRE1α (grey) and after incubation with Mg/ATP (black). **(C)** 5’FAM, 3’BHQ-labeled XBP-1 splice site mimic RNA was incubated with increasing concentrations of phosphorylated GST-G547 IRE1α (filled triangles) or dephosphorylated GST-G547 IRE1α (open triangles) for 30 minutes at 30°C. Error bars represent the standard error of the mean of 3 independent experiments. **(D)** Comparison of extent of cleavage of the labelled XBP1 splice site mimic RNA when incubated with 100 nM of the indicated IRE1α construct, with or without pre-incubation with ATP, for 1 hour at 30°C, error bars show the S.E.M of three independent experiments. **(E)** Immunoblot of H499 IRE1α after treatment with 250 μM of the crosslinker disuccinimidyl suberate (DSS). Increasing concentrations of either dephosphorylated or autophosphorylated H499 IRE1α were incubated with DSS for 45 minutes before separation by SDS-PAGE and transfer to nitrocellulose membrane. Monomer* indicates the reduced mobility of autophosphorylated H499 IRE1α. **(F)** Comparison of phosphorylation status of IRE1α immunoprecipitated from human cells and purified IRE1α constructs. Phosphorylation sites of IRE1α were determined by tryptic digest mass spectrometry in protein immunoprecipitated from human cells (Cell IP), autophosphorylated G547 IRE1α (G547) and autophosphorylated H499 IRE1α (H499). Where the site could not be uniquely identified a ‘/’ is used to indicate the possible residues. *T973 discovered in immunoprecipitated IRE1α when chymotrypsin was used for digestion. Only trypsin was used to digest H499 and G547.

The experiments above demonstrate that IRE1α can be activated in the absence of phosphorylation which we hypothesised was caused by concentration-dependent oligomerisation. To test this hypothesis we investigated whether constitutively dimerised G547 IRE1α would be more active than monomeric G547 IRE1α. Dimerisation was achieved by fusion of the cytosolic domain to glutathione S-transferase (GST) and confirmed by native gel electrophoresis (Figure 2A). Protein was dephosphorylated and confirmed as before (Figure 2B, (Additional file 1: Figure S1). GST-G547 IRE1α was incubated with Mg/ATP to generate the autophosphorylated species, and as for G547 IRE1α, the GST-G547 IRE1α was tri-phosphorylated. Notably, there was an increase in mass of 2 amu suggesting a disulfide bridge reduction in this protein sample.

In the FRET derepression assay, fully dephosphorylated GST- G547 IRE1α reached maximal activity at much lower concentrations than either phosphorylated or dephosphorylated G547 IRE1α, with ~70% of full activity achieved at 25 nM, the lowest concentration tested, (Figure 2C). These data support the hypothesis that human IRE1α cytosolic domain can be activated by constitutive dimerisation. However, at low concentrations, phosphorylated GST-G547 IRE1α had a higher activity than the dephosphorylated GST- G547 IRE1α indicating that phosphorylation still enhances the activity of a constitutive dimer (Figure 2C). Two hypotheses can be proposed to explain these results; phosphorylation may further stabilise/enable an RNase-competent dimeric complex of the cytosolic domains. Alternatively, phosphorylation may lead to local conformational changes in each monomer that enhance intrinsic catalytic activity. Crystallisation of human IRE1α with XBP1 mRNA or with a suitable non-cleavable analogue of XBP1 mRNA bound is eagerly awaited to confirm these hypotheses.

### Autophosphorylation enhances stability of IRE1α dimers

Having shown that dimerisation causes increased activity, we predicted that the increased RNase activity of autophosphorylated H499 IRE1α relative to dephosphorylated H499 IRE1α is caused by enhanced stability of dimers in the autophosphorylated state. To test this, we incubated increasing concentrations of fully dephosphorylated H499 IRE1α and autophosphorylated H499 IRE1α with the crosslinking agent disuccinimidyl suberate and probed for the presence of monomers and dimers/oligomers by western blot (Figure 2E). Dephosphorylated H499 IRE1α was present mainly in monomeric form, with small amounts of dimer at high concentration, whereas autophosphorylated H499 IRE1α formed dimers and oligomers at the lowest tested concentration of 0.5 μM. Similar results were seen for G547 IRE1α (data not shown). Taken together with our RNase activity data (Figure 1G), these data support a model in which autophosphorylation enhances RNase activity by stabilising the dimeric/oligomeric form of IRE1α.

### Hyperphosphorylation of IRE1α is located in the activation loop and linker domain

As H499 IRE1α is hyperphosphorylated relative to G547 IRE1α, we proposed that the linker domain of human IRE1α may serve as a hyperphosphorylation site for the Ser/Thr kinase activity of IRE1α. To localise the sites of phosphorylation, autophosphorylated H499 IRE1α and G547 IRE1α were analysed by tryptic digest mass spectrometry. It was not possible to uniquely assign the phosphorylation sites in all of these peptide fragments due to the density of serine and threonine residues in the linker and activation loop sequences, however, spectra revealed that both H499 IRE1α and G547 IRE1α were phosphorylated on the activation loop at residues Ser724, Ser726 and Ser729/Thr734 (Figure 2F). Phosphorylation at Thr884 in the RNase domain was also seen, as was a shared phosphorylation site in the part of the linker region common to both constructs at Ser548/Ser549/Ser551. G547 IRE1α had an additional phosphorylation in the linker domain, not seen in H499 IRE1α, at Thr561/Ser562. Of the additional phosphorylations present in H499 IRE1α one double phosphorylation site was located to the extended linker region between S533-S539. Six additional phosphorylations were also spread throughout the protein (Figure 2F). We speculate that the additional phosphorylations we detected spread throughout the protein may be low level phosphorylations. Hyperphosphorylation in the linker may have also occurred but was not detected by mass spectrometry due to the limitations of the technology to detect heavily phosphorylated peptides.

### Hyperphosphorylation of IRE1α also occurs in vivo

To ascertain if hyperphosphorylation of the linker domain was an artefact of *in vitro* autophosphorylation or relevant *in vivo*, IRE1α was activated in H929 myeloma plasma cells using tunicamycin to induce proteotoxic stress. IRE1α was immunoprecipitated from cells and subjected to tryptic digestion and mass spectrometry. Four phosphorylated peptide fragments corresponding to 6 phosphorylation sites were observed. These were located in; the extended linker at S548/S549/S551, the activation loop at both S724 and S726 and additionally at T973 in the C-terminus (Figure 2F).

These data demonstrate that hyperphosphorylation of IRE1α, particularly in the activation loop and linker domain, also occurs in cells, and confirms that phosphorylations seen *in vitro* are relevant *in vivo*.

## Conclusions

These data strongly support a model where RNase activity is achieved through dimerisation and clearly show that multiple autophosphorylations enhance the RNase activity of human IRE1α *in vitro* through stabilising dimerisation. *In vivo*, human IRE1α is a transmembrane protein whose stress-sensing luminal domain is negatively regulated by the ER-resident chaperone BiP [14]. *In vitro*, without the regulatory luminal domain, without hindrance to oligomerisation of the cytosolic domain, stochastic formation of oligomers will increase in line with protein concentration leading to increased endonuclease activity [16]. Therefore, the systems used in this paper and others do not fully reconstitute the activation process in cells. However, we speculate that results obtained here mimic the behaviour of IRE1α when BiP is not bound and provide insight to the behaviour of full-length IRE1α. Based on these data we suggest that the development of IRE1α kinase inhibitors could prevent RNase activity and subsequent splicing of XBP1 which would be of potential therapeutic use for the treatment of cancer.

## Methods

### Cloning, expression, and purification of human IRE1α

G547-L977 and H499-L977 IRE1α proteins were prepared by expression in Sf9 insect cells essentially as described [25] with purification over a Mono-Q column replaced by purification over a 6 ml Resource™ Q column (GE healthcare, Waukesha, USA) equilibrated in 50 mM Hepes pH7.5, 20 mM NaCl, 1 mM EDTA, 1 mM DTT, 10% glycerol and eluting using an NaCl gradient. For GST-tagged protein, G547-L977 was inserted into a modified version of pFastBac1 encoding an N-terminal 6xHis tag followed by GST tag and human rhinovirus 3C protease site. Following purification over Talon resin (Clontech, Mountain View, USA), His-GST tagged protein, was purified over a 5 ml GSTrap™ FF column (GE healthcare, Waukesha, USA), equilibrated in 50 mM Hepes pH7.5, 300 mM NaCl, 2 mM DTT, 1 mM EDTA, 10% glycerol, eluting with 20 mM glutathione. His-GST-G547 IRE1α was further purified by size exclusion chromatography on a Superdex200 column (GE Healthcare).

### In vitro autophosphorylation

10 μM IRE1α was incubated with 5 mM ATP and 25 mM MgCl_2_ (Sigma) at 30°C for 1 hour in buffer containing 50 mM Hepes pH7.5, 120 mM NaCl, 2 mM DTT, 1 mM EDTA and 10% glycerol. Protein was purified from excess MgCl_2_ and ATP using Zeba™ spin desalting columns according to the manufacturer’s instructions (Thermo scientific).

### High resolution LC/MS analysis of intact IRE1α proteins

Mass spectrometry analysis of the intact protein was carried out after desalting and buffer removal using a Phenomenex Security Guard C8 column cartridge. Methodology for separation and analyses are provided in Additional file 2.

### FRET derepression assay

Experiments were performed in 96-well format, in triplicate and set up on ice before incubation. Reactions were run in cleavage buffer: 20 mM HEPES pH7.5, 50 mM KOAc, 0.5 mM MgCl2, 3 mM DTT and 0.4% PEG-400. IRE1α was added first, followed by addition of 90 nM fluorescence quenched XBP1 RNA cleavage site mimic 5’FAM GAACAAGAUAUCCGCAGCAUAUACAGUUC 3’BHQ (Eurofins MWG Operon). Plates were incubated at 30°C for 30 minutes and fluorescence readings taken on a 7500 Fast Real-time PCR system (Applied Biosystems). Fluorescence measurements were converted into RNA concentrations by use of a standard curve; created by incubating increasing concentrations of substrate with RNase A.

### IRE1α crosslinking

Increasing concentrations of protein were crosslinked by incubation with 250 μM disuccinimidyl suberate (Sigma) for 45 minutes at room temperature in buffer containing 50 mM Hepes pH7.5, 120 mM NaCl, 2 mM DTT, 1 mM EDTA and 10% glycerol. The crosslinking reaction was quenched with 50 mM Tris–HCl pH 7.5. Samples were subjected to electrophoresis on a NuPAGE 4-12% Bis-Tris gel (Life technologies) and immunoblotted using an anti-IRE1α antibody (Cell Signalling Technologies).

### Immunoprecipitation of IRE1α from myeloma cells

NCI-H929 cells were treated with 10 μg/ml tunicamycin for 4 hours. Cells were lysed in buffer containing 1% Triton X-100; 10 mM Tris pH7.6; 10 mM EDTA; 150 mM NaCl and twice standard concentrations of PhosStop and complete protease inhibitors (Roche). Lysates were spun at 12000 × g for 10 minutes to remove nuclei. Supernatants were transferred to fresh tubes and incubated overnight with anti-IRE1α antibody (Cell Signaling Technology, Danvers, MA, USA). Antibody:IRE1α complexes were captured with protein A/G magnetic beads (Thermo Fisher Scientific, Hemel Hempstead, UK) and washed five times in lysis buffer before elution into reducing Laemmli sample buffer. Proteins were separated by SDS-PAGE and stained with coomassie for mass spectrometry.

### Protein digestion, LC-MS/MS analysis and database interrogation

Procedures were performed as previously described [26] except that for MS a 1.7 kV ionisation voltage was applied and multistage activation was used in place of wideband activation to co-fragment phosphate neutral losses of 32.70, 49.00, 65.40 and 98.00 *m/z* from phosphopeptide precursor ions, if observed in the top 3 most intense fragment ions. H499 and G547 samples were analysed using a packed emitter LC setup as previously described [27], and with the MS dynamic exclusion reduced to 10 s. Database interrogation differed as follows: precursor ion tolerance 5 ppm, MS/MS fragment tolerance 0.25 Da, interrogation against the swissprot 2011_01 database customized to include the IRE1 construct sequences, and phosphorylation(STY) added as a variable modification.

## Competing interests

All authors are employees of The Institute of Cancer Research which has a commercial interest in the development of inhibitors of the stress response. The authors declare that they have no competing interests.

## Authors’ contributions

DI carried out the RNase assays, participated in the design of the study and drafted the manuscript. MB performed the immunoprecipitation experiment. PM made the protein constructs and participated in the design of the study. AM performed the intact mass spectrometry and analysis. YN and JS participated in data analysis. MW and AT performed tryptic mass spectrometry and analysed data. GM, IC and FD conceived of the study, and participated in its design and coordination and helped to draft the manuscript. All authors read and approved the final manuscript.

## Supplementary Material

Additional file 1: Figure S1 Phosphorylation status of purified IRE1α constructs before and after in vitro autophosphorylation. Click here for file

Additional file 2 Supplementary methods. Click here for file
