# Supplementary figures and images for: Multiple autophosphorylations significantly enhance the endoribonuclease activity of human inositol requiring enzyme 1α
Source: BMC Biochem. 2014 Feb 13;15:3. doi: 10.1186/1471-2091-15-3 (PMC3928614; doi:10.1186/1471-2091-15-3)

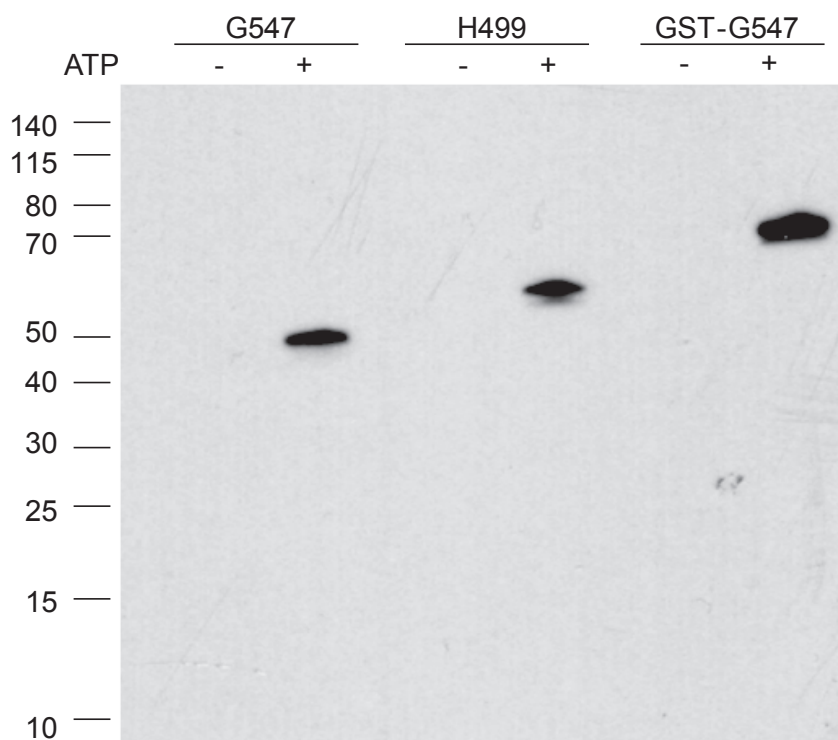

Figure S1.

Supplement: Additional file 1: Figure S1 — Phosphorylation status of purified IRE1α constructs before and after in vitro autophosphorylation. [file 1471-2091-15-3-S1.pdf]
