# Supplementary material for: Multiple autophosphorylations significantly enhance the endoribonuclease activity of human inositol requiring enzyme 1α
Source: BMC Biochem. 2014 Feb 13;15:3. doi: 10.1186/1471-2091-15-3 (PMC3928614; doi:10.1186/1471-2091-15-3)
Supplement: Additional file 2 — Supplementary methods. [file 1471-2091-15-3-S2.doc]

**SUPPLEMENTAL MATERIAL TO:**

Multiple autophosphorylations significantly enhance the endoribonuclease activity of human Inositol Requiring Enzyme 1

**Daniel N. Itzhak^1,2^, Michael D. Bright^1,2^, Peter C. McAndrew^1^, Amin Mirza^1^, Yvette Newbatt^1^, Jade Strover^1^, Marcella Widya^3^, Andrew J. Thompson^3^, Gareth J. Morgan^1^, Ian Collins^2^ and Faith E. Davies^1,2^**

^1^ From the Division of Cancer Therapeutics, Institute of Cancer Research, Sutton, Surrey, SM2 5NG, UK

^2^ Division of Molecular Pathology, Institute of Cancer Research, Sutton, Surrey, SM2 5NG, UK

^3^ Proteomics Core Facility, Institute of Cancer Research, London, SW3 6JB, UK

**Supplemental Figure S1**. Phosphorylation status of purified IRE1α constructs before and after in vitro autophosphorylation. λ-phosphatase treated and purified IRE1α containing; 24 amino acids of the linker domain (G547), 72 amino acids of the linker domain (H499) or 24 amino acids of the linker domain plus an N-terminal GST-tag (GST-G547), were incubated with 25mM Magnesium Chloride with or without 5mM ATP, as indicated. Protein samples were purified as described in the experimental procedures and analyzed by SDS-PAGE followed by western blot with an antibody specific for phospho-Ser724.

**Supplementary methods**

*Western Blotting-* Determination of IRE1α phosphorylation status was performed as previously described (25). An anti-phospho Ser724 antibody (Abcam) (1/2000) dilution was used to detect phospho-Ser724, horseradish peroxidase-conjugated secondary antibody at 1/5000 was added after washing and bands were visualised using ECL chemiluminescent reagents (GE Healthcare) and developed on Biomax film (Kodak).

*Processing and analysis of LC-MS/MS data-*Raw MS/MS data were compiled into peaklists using Proteome Discoverer v1.3 (Thermo Fisher Scientific, Hemel Hempstead, UK) default parameters. Peaklists were interrogated against the SwissProt database 2011_01 using Mascot v2.2 (www.matrixscience.com) with *homo sapiens* taxonomy filter (20,305 sequences) and assuming tryptic enzyme specificity with up to two missed cleavages, or against a SwissProt 2011_01 *homo sapiens* subset database (20,282 sequences) customised to include the IRE1 construct H499 and G547 sequences. A precursor ion tolerance of 5 ppm and fragment ion tolerance of 0.25 Da was applied and the following variable modifications were accounted for: acetylation of the protein N-terminus, carbamidomethylation of cysteine, oxidation of methionine, pyroglutamisation of peptide N-terminal glutamine and phosphorylation of serine, threonine and tyrosine. The Mascot peptide and protein identification results were grouped and validated using Scaffold v3.6 (Proteome Software Inc., Portland, OR). Protein identifications were automatically accepted if they contained at least 2 unique peptides assigned with at least 95% confidence by Peptide Prophet . To identify phosphorylation sites, the peptide confidence threshold was reduced to 50% and potential phosphopeptide spectra were visually assessed.

**References:**

1. Keller, A., Nesvizhskii, A. I., Kolker, E., and Aebersold, R. (2002) Empirical statistical model to estimate the accuracy of peptide identifications made by MS/MS and database search*. Anal Ch*e**m** 74, 5383-539
